# State-of-the-art micro- and nano-scale photonics research in Asia: devices, fabrication, manufacturing, and applications

**DOI:** 10.1038/s41378-024-00736-y

**Published:** 2024-08-22

**Authors:** Hyunjung Kang, Takuo Tanaka, Huigao Duan, Tun Cao, Junsuk Rho

**Affiliations:** 1 Department of Mechanical Engineering, Pohang University of Science and Technology (POSTECH), Pohang, 37673 Republic of Korea; 2 Innovative Photon Manipulation Research Team, RIKEN Center for Advanced Photonics, Wako, Saitama, 351-0198 Japan; 3 Metamaterials Laboratory, RIKEN Cluster for Pioneering Research, Wako, Saitama, 351-0109 Japan; 4 Institute of Post-LED Photonics, Tokushima University, Tokushima, 770-8506 Japan; 5 National Research Center for High-Efficiency Grinding, College of Mechanical and Vehicle Engineering, Hunan University, Changsha, 410082 China; 6 Greater Bay Area Institute for Innovation, Hunan University, Guangzhou, 511300 China; 7 School of Optoelectronic Engineering and Instrumentation Science, Dalian University of Technology, Dalian, 116024 China; 8 Department of Chemical Engineering, Pohang University of Science and Technology (POSTECH), Pohang, 37673 Republic of Korea; 9 Department of Electrical Engineering, Pohang University of Science and Technology (POSTECH), Pohang, 37673 Republic of Korea; 10 POSCO-POSTECH-RIST Convergence Research Center for Flat Optics and Metaphotonics, Pohang, 37673 Republic of Korea

**Keywords:** Nanophotonics and plasmonics, Nanoscale devices, Materials science

**Fig. 1:**
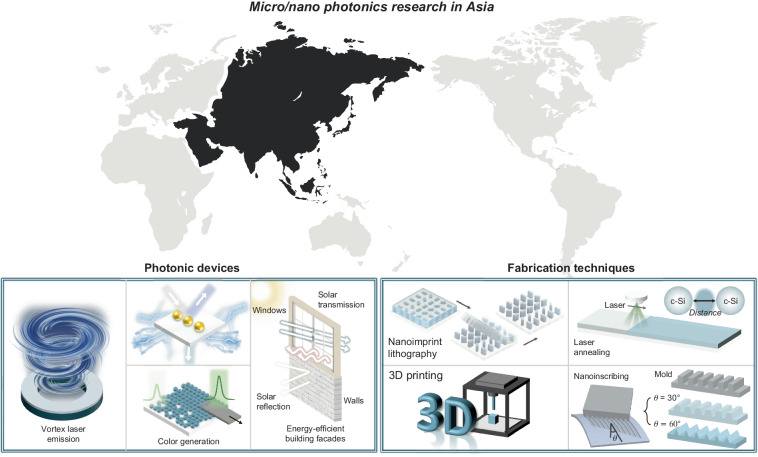
Micro- and nano-scale photonics research in Asia. Sub-micro-scale research, including laser emission, displays, building facades, and various fabrication techniques, has been actively conducted by leading research communities in Asia

Micro- and nano-scale photonics research has gained significant attention, offering various applications from process development to imaging platforms and augmented reality displays^1–4^. In Asia, this research is advancing rapidly, positioning the region as a leading force in global photonics innovation. This special issue is dedicated to highlighting state-of-the-art developments in micro- and nano-scale photonics, devices, fabrication, manufacturing, and applications, with a focus on the valuable contributions from top researchers in the region. In recent years, Asian countries have solidified their status as global leaders by investing heavily in cutting-edge research facilities and fostering robust collaborations between universities, research institutions, and industries^5^. These combined efforts have propelled the region to the forefront of technological advancement, with various applications with the potential to transform multiple sectors and improve the quality of life globally. This special issue presents the latest advancements in optical devices, fabrication, manufacturing, and applications in micro- and nano-scale photonics research within Asia, covering a wide range of topics (Fig. 1). We explore color generation^6,7^, laser emission^8^, and nano-fabrication techniques along with their applications^9–13^. Additionally, we introduce recent developments in fabrication methods utilizing magnetic cilia^14^, hydrogels^15^, and energy-efficient bulding facades^16^. We hope this special issue will serve as a valuable reference for current and future research directions in sub-micro-scale photonics, demonstrating the remarkable advancements made in Asia and inspiring continued innovation and collaboration in this dynamic and rapidly evolving field.

Color generation techniques are important for realizing high-resolution imaging and displays with low power consumption and compact device size^17^. However, pigment-based color printing has limitations such as low resolution and durability, with colors fading over time. Alternatively, nature demonstrates that colors can be generated through micro- and nano-scale structures, which can be engineered through diffractive optics, photonic crystals, and plasmonics. These structures produce colors by scattering or partially absorbing light within the structured materials. One strategy involves multicolor nano-filters consisting of multilayered ‘active’ plasmonic nanocomposites, where metallic nanoparticles are embedded within a conductive polymer nanofilm^6^. Such nanocomposites have been fabricated at the wafer scale with a total thickness below 100 nm using a “lithography-free” method. They inherently exhibit three prominent optical modes, accompanied by scattering phenomena that produce distinct dichroic reflective and transmissive colors. The electrical manipulation of color over the entire visible spectrum has been demonstrated with fast switching speeds, offering potential applications in various electronic devices, ranging from personal devices to public management systems. Recently, hyperreflective photonic crystals have been demonstrated through colloidal crystallization^7^. To improve the uniformity and reproducibility, shear flow is applied to the dispersions, causing silica particles to rearrange into larger crystalline domains with a unidirectional orientation along the direction of the flow. This shear-induced structural change achieves an absolute reflectivity of 90% in the stop band, and a high transparency of 90% at off-resonant wavelengths. These innovative approaches hold great potential for advancing color filtering and display technologies.

Modern optical communication based on orbital angular momentum (OAM) has attracted significant attention as a way to enhance the channel information capacity. OAM beams, defined by a topological charge, can encode information in classical and quantum systems due to the orthogonality of different modes. Optical vortices carrying OAM have shown promise in improving spectral efficiency. A novel approach for tunable vortex lasing has recently been demonstrated, using a micro-ring cavity integrated with the phase change material Ge_2_Sb_2_Te_5_ (GST225)^8^. This technique allows for the tuning of the resonant wavelength while maintaining a consistent toroidal intensity distribution. By adjusting the complex refractive index to achieve an exceptional point (EP), the microlaser generates artificial angular momentum and emits vortex beams with precise OAM. The wavelength of the vortex laser from the micro-ring cavity can be dynamically adjusted by switching the state of GST225 between amorphous and crystalline forms. This tunable OAM microlaser opens new possibilities for applications in OAM multiplexing, optical trapping, and optical communications.

Micro- and nano-fabrication techniques are at the forefront of modern technological advancements, enabling the precise manipulation and structuring of materials at extremely small scales. These techniques play a crucial role in a wide range of applications, including electronics, photonics, biomedical devices, and energy solutions. The increasing interest in this field is driven by the need for miniaturization and the development of devices with enhanced performance and novel functionalities. Laser annealing for silicon nanoparticles has been proposed as a straightforward and efficient fabrication method^9^. These nanoparticles exhibit Mie resonances in the visible spectrum, with precisely controllable resonant wavelengths. A significant outcome of this method is a 60-fold enhancement in fluorescence, highlighting its potential for highly sensitive fluorescence sensing and biomedical imaging applications. Recently, azimuthal-rotation-controlled dynamic nanoinscribing (ARC-DNI) process has offered a continuous and scalable approach to fabricating asymmetric nanograting structures with tunable periods and shapes^10^. By adjusting the azimuthal angle and other parameters such as temperature, force, and inscribing speed, the ability to create diverse nanograting profiles, including trapezoidal, triangular, and parallelogrammatic shapes has been demonstrated. This versatility makes ARC-DNI suitable for fabricating various optical devices, as exemplified by asymmetric diffractive optical elements. Additionally, to address the challenges of high cost and low throughput in optical metasurface manufacturing, high-refractive-index zirconium dioxide (ZrO_2_) nanocomposites in nanoimprint lithography (NIL) has been proposed^11^. By optimizing the composition of ZrO_2_ nanoparticle concentrations and solvents, high conversion efficiencies for ultraviolet metaholograms have been achieved. This advancement enhances the practical applicability of optical metasurfaces, making NIL a valuable tool in the field. Furthermore, nanoimprint-induced strain engineering for 2D materials has been presented as a novel method for generating controllable periodic strain profiles in 2D materials, such as molybdenum disulfide^12^. By pressing the material against an imprint mold, different strain profiles are created and verified using Raman and photoluminescence spectroscopy. This technique highlights the ability to precisely control strain magnitudes and distributions, offering a deterministic approach to strain engineering that is compatible with standard semiconductor fabrication processes. 3D printing has emerged as a transformative technology for fabricating energy devices with complex 3D structures^13^. This technique allows for the creation of micro-lattice structures that enhance both mechanical properties and electrical performance compared to bulk counterparts. With the advancement of 3D printing processes, 3D-printed energy devices with enhanced mechanical property, integrability, high resolution, and exceptional electrical performance will eventually find widespread use across various fields.

To implement a wide range of applications such as photonic devices and sensors, various materials and fabrication methods are being extensively researched. In particular, micro- and nano-scale cilia, which serve diverse biological functions in natural systems, have inspired the development of artificial magnetic cilia^14^. These biomimetic systems that utilize various magnetic particles hold significant potential in soft robotics, droplet and particle control systems, fluidics, optical devices, and high-precision sensors. Their fabrication involves both top-down and bottom-up techniques, ensuring accessibility without being limited by specific processes. Additionally, hydrogels have emerged as a promising field in active photonics, providing deformable geometric parameters in response to external stimuli such as humidity^15,17^. Recent advancements in hydrogels have focused on the development of stimuli-responsive photonic devices with tunable optical properties. Key micro- and nano-fabrication techniques for hydrogel-based photonic devices include film growth, photolithography, electron-beam lithography, and NIL. Emerging technologies utilizing magnetic cilia and hydrogels will advance the field, including nanomaterials, innovative 3D manufacturing, flexible electronics, and artificial intelligence, with unprecedented functionality.

Despite rapid advancements in clean energy technologies, buildings still account for a significant portion of global energy consumption and carbon emissions, primarily due to heating, ventilation, and air conditioning systems. Energy-efficient buildings offer a promising solution to reduce energy usage by engineering windows, walls, and roofs to manage heat transfer through electromagnetic radiation by controlling solar irradiation and thermal emission properties^16^. For example, smart windows, which employ dynamic chromogenic materials to modulate the sunlight transmitted to the indoors, can significantly reduce energy consumption for heating and cooling. Advanced fabrication methods, including coating, vapor deposition, nanolithography, printing, etching, and electrospinning, are crucial in developing such energy-efficient materials. The latest developments in these techniques hold promise for enhancing the design and performance of energy-efficient buildings.

In conclusion, the research presented in this special issue highlights Asia’s leading role in advancements in micro- and nano-scale photonics. The innovations in photonics technology^6–8^ and micro- and nano-scale fabrication techniques^9–13^ have the potential to advance various fields, including communications, energy, and materials science. Additionally, the development of fabrication methods for magnetic cilia^14^, hydrogels^15^, and energy-efficient materials^16^ enhances device performance and sustainability. It also introduces novel functionalities with potential for advancements in research and technology. The advancements introduced in this special issue will influence global technological progress, leading to more real-world applications.
